# Supplementary material for: High-order tensor flow processing using integrated photonic circuits
Source: Nat Commun. 2022 Dec 28;13:7970. doi: 10.1038/s41467-022-35723-2 (PMC9797566; doi:10.1038/s41467-022-35723-2)
Supplement: Supplementary file 1 — Supplementary Information [file 41467_2022_35723_MOESM1_ESM.pdf]

Supplementary Information for

High-order tensor flow processing using integrated photonic circuits

Shaofu Xu[1†], Jing Wang[1†], Sicheng Yi[1], and Weiwen Zou[1]*

[1]State Key Laboratory of Advanced Optical Communication Systems and Networks, Intelligent Microwave Lightwave Integration Innovation Center (imLic), Department of Electronic Engineering, Shanghai Jiao Tong University, 800 Dongchuan Road, Shanghai 200240, China

[†]These authors contribute equally to this work

**Correspondence to: wzou@sjtu.edu.cn*

This PDF file includes:

Supplementary Notes 1 to 7
Supplementary Figs. 1 to 23
Supplementary Tables 1 to 5
Supplementary References 1 to 14

Supplementary Note 1 - Directional couplers for light splitting

The Si_3N_4 directional coupler is utilized to share power equally for weighting. Suppl. Fig. 3a shows the schematic of the directional coupler, which consists of two narrow Si_3N_4 waveguides, one S-bend waveguide, and one 90° arc-bend waveguide. The two separating waveguides are close so that the evanescent fields couple their optical modes. Suppl. Fig. 3b shows a cross-section of the narrow Si_3N_4 waveguide. Suppl. Table 1 shows the design parameters labeled in Suppl. Fig. 3a and 3b. Suppl. Fig. 3c shows the directional coupler array (DCA) which is cascaded by directional couplers with different splitting ratios. The splitting ratio between the through port and cross port can be adjusted by varying the coupling length. The splitting ratio versus the coupling length at 1550 nm and the wavelength dependence of the splitting ratio are shown in Suppl. Fig. 4. Here, the lengths of the directional couplers are concluded in Suppl. Table 2, which are selected according to the splitting ratio at 1550 nm. The measured loss of the DCA is shown in Suppl. Fig. 5. The maximum difference of the loss between the cross ports and the through port is around 8 dB.

Supplementary Note 2 - MRR weighting banks

The tunable Si microring weighting bank provides individual weighting to the input light of different wavelengths. Suppl. Figs. 6a and 6b show the schematic of the single MRR and the cross section, respectively. The MRR can be tuned by micro heater. Suppl. Fig. 6c shows the schematic of the MRR weighting bank. The Si weighting bank consists of four MRRs coupled with two bus waveguides (the input port and output port with the lower bus waveguide are capable of weighting, and the monitoring port with the upper bus waveguide is used for characterizing the weighting bank). The four MRs have radii of 10.00 μm, 10.02 μm, 10.04 μm, and 10.06 μm, respectively. The other parameters of the weighting bank including the coupling gap and the waveguide width are concluded in Suppl. Table 3. Suppl. Fig. 7a shows the calculated transmission and the phase responses of the tunable Si micro-ring weight bank. The transmission response of the MRR weighting bank can be controlled by the micro heater. Consequently, the shift of resonant wavelength $\Delta\lambda$ is proportional to the variation in temperature ΔT, which can be described as

$$\Delta\lambda = -\Delta T \cdot \frac{\lambda_0}{n} \cdot \left(\frac{\Delta n}{\Delta T}\right) \tag{1}$$

where λ_0 is the resonance wavelength of the micro-ring resonator, Δn is the effective index, and $\Delta n/\Delta T$ is the thermo-optic coefficient of Si (1.86×10^{-4} K^{-1}). Suppl. Fig. 7b shows that with increasing heating power, the resonant wavelength presents a linear shift with a tuning efficiency of 78.9 pm/mW. To characterize the practical performance of the MRR weighting bank, an experimental setup is exploited (shown in Suppl. Fig. 8), where an amplifier spontaneous emission (ASE) light source is used to measure the transmission spectrum between monitoring port and output port. Results are shown in Fig. 2f and 2g in the main text.

Supplementary Note 3 - Tunable Si_3N_4 wavelength division multiplexer

The wavelength division multiplexing (WDM) is utilized to speed up the processing by transferring multiple wavelength carriers. The Si_3N_4 WDM consists of the asymmetric Mach–Zehnder interferometers in a binary tree [1]. Suppl. Fig. 9a shows the schematic of the asymmetric MZI. As can be seen, the MZIs are based on 2×2 directional couplers and phase shifters. The lower phase shifters consist of the narrow waveguides and the tapers. The upper phase shifters consist of the tapers and two kinds of waveguides: the narrow waveguides and the wide waveguides. The narrow waveguides are introduced to realize 180° bends. The wide waveguides are introduced to realized fabrication-robust MZIs, since the width variation can lead to phase error. The length of the wide waveguide is denoted as ΔL, and the pure optical path length difference of the two arms in phase shifters is contributed by the length of the wide waveguides.

Suppl. Fig. 9b shows the cross section of the wide waveguide with the micro-heater, which is introduced for thermal tunning. The waveguide taper between the wide waveguide and the narrow waveguide is shown in Suppl. Fig. 9c. Suppl. Fig. 9d shows the 4-channel MZI-type WDM. To achieve the power cross-coupling coefficients of around 0.5, 0.29 and 0.08 [1], the coupling lengths of the directional couplers with the edge-to-edge coupling gap fixed at 0.45 μm are selected as 15.55 μm, 8.94 μm and 0.30 μm, respectively. The parameters of the MZI-type WDM are concluded in Suppl. Table 4. All the bends in this device have the same radius.

Suppl. Fig. 10a shows the calculated transmission and phase responses of the 4-channel WDM. The transmission response of the Si_3N_4 MZI can be controlled by the micro heater, and the thermal sensitivity can be described as Eq. (1). The simulated wavelength shift is shown in Suppl. Fig. 10b. As seen, the thermal tuning efficiency is 5.6 pm/W. The measured results of the fabricated WDM are shown in Fig. 2d in the main text, which agree well with the calculated results.

Supplementary Note 4 - Si_3N_4 delay line

Optical delay line, through which the light is delayed by a certain amount of time. Suppl. Fig. 11a shows the schematic of the Si_3N_4 delay line. The wide waveguides which support multi-mode are utilized to reduce the waveguide loss by suppressing the scattering loss. The 90° Euler bends are utilized to suppress the inter-mode crosstalk of the multi-mode waveguides [2]. The adjacent waveguides are separated by 10 μm (edge-to-edge), and the maximal and minimal curvature radii (R_{max} and R_{min}) of the 90° Euler bend are selected as 1000 μm and 60 μm, respectively. The effective radius (R_{eff}) is approximately 106 μm.

The length of the delay line is determined by the certain waveguide's group delay, which can be calculated as:

$$L = \frac{c\tau}{n_g} \tag{2}$$

where c is the speed of light in vacuum, τ is the group delay, and n_g is the group index. n_g at 1550 nm is 2.0189. Considering the certain group delay is 100 ps, we choose 14849.65 μm as the length of the Si_3N_4 delay line. Suppl. Fig. 12a shows the wavelength dependency of the group delay. Suppl. Fig. 12b shows the simulated transmission from the fundamental TE mode launched at the input to the high order TE mode in the output. The transmission losses from TE0 mode to TE0 mode are < 0.01 dB and the inter-mode crosstalk TE0 mode to TE_i mode (i = 1, 2) is < −30 dB over 1540~1560 nm.

Suppl. Fig. 13a shows the measured transmission spectrum of the Si_3N_4 delay lines over 1520~1600 nm. Here, we characterized two kinds of delay line including the narrow waveguides with the width of 1 μm and the wide waveguides with the width of 3 μm. The waveguide losses are around 5.1 dB/cm and 1.5 dB/cm, shown in the Suppl. Fig. 13b.

Supplementary Note 5 - Insertion loss of the PTFP chip

The insertion loss of the chip is mainly composed of several parts: fiber-chip edge coupling, WDM, directional coupler splitters, MRRs, and the waveguide loss from the input to the output of the PTFP chip. We take the insertion loss at 1550 nm as the typical value. The insertion loss of fiber-chip edge coupling is around 6.5 dB/facet. It is ~5 dB for the WDM. Although the insertion loss of the directional coupler itself is very low and negligible, the unevenness of the light splitting

results in the same effect as insertion loss. The optical power should be identical for different optical paths, otherwise the weights of computing will be incorrect. In this case, we should choose the minimal optical power to represent the weight 1, and attenuate other optical powers to the same level as the minimal one to represent the same weight 1. Consequently, the 8-dB unevenness of the directional coupler light splitter introduces 8-dB insertion loss in maximum. The unevenness of the DCA (a test device with 8 cascaded couplers) in Supplementary note 1 is measure at 8 dB. However, in the PTFP chip, a DCA with 2 cascaded couplers is used. The unevenness of the adopted DCA is measured at ~3 dB. Combing the insertion loss of the optical delay line (~6 dB for the length of 4 cm), the total unevenness of the optical delay structure is ~8 dB. In summary, the maximal insertion loss of the current version of PTFP chip is ~29 dB. To compensate for this large loss, we adopted optical amplification (the EDFAs) and electrical amplification (the power amplifier) in the experiment. However, these amplifiers not only increase system complexity and power budget, but also introduce some unnecessary processes such as encoding and decoding. Therefore, in future engineering, the insertion loss of the PTFP chip should be refined significantly from following aspects: using advanced fiber-chip coupling; using low-loss delay lines; using low-loss WDM; increasing the evenness of DCA; using on-chip PD to avoid chip-fiber coupling.

Supplementary Note 6 - Future scaling of the PTFP chip

In the proof-of-concept experiment, the PTFP chip comprises the dimensionality of [D_w =4, D_t =3, D_s =1]. There is a large space to augment the scale of the chip. Large-scale integration of PTFP will lead to high-order processing capability, high throughput, and efficiency of computing.

The wavelength dimension can be augmented. The feasible number of wavelengths is dependent on the quality factor of MRRs and the width of the free spectral range. According to [3], the capable number of wavelengths in a weighting bank can theoretically reach 131.

The space dimension can be extended by simply duplicating the same structure of MRR weighting banks for multiple copies. The limit of the space dimension is the signal-to-noise ratio (SNR) and the factors behind SNR. Since the optical signals of all space dimensions come from the WDM, the optical intensity is limited by the nonlinear effects of the output waveguide of the WDM. With limited optical power, increasing spatial duplication leads to low optical power in each copy. Given that the noise of amplified photodetectors is partly independent of the input optical power, the SNR is lowered with a larger spatial dimension. In [4], the maximum spatial

splitting is evaluated. Using Si_3N_4 waveguides, when the insertion loss (additional loss excluding the theoretical splitting loss) of the optical link is less than 7.4 dB, the space dimension can reach 32 for 3×3 kernels. The optical SNR is maintained higher than 10 dB. Optical SNR higher than 10 dB means the SNR of the converted electrical signal is higher than 20 dB, which is sufficient for most classification tasks. Note that the evaluated photodetector in [4] is commercially available ones. When advanced photodetectors such as high-speed avalanche PDs are adopted, the SNR can be increased further.

Currently, the fabricated chip comprises 3 delay steps. By augmenting the number of delay steps, the PTFT can process larger convolutional kernels without data duplication. One way is to cascade more ODLs with the same length. It can enlarge the kernel size of one-dimensional convolution. The other is to introduce long ODLs to form higher-dimensional convolutions. Take two-dimensional image convolution ($\sigma\times\sigma$) for example. If the image is input row by row, the required delays are not uniform. For an image sized $L\times L$, the required delay for q-th time dimension is defined as [5]:

$$D_q = \left[\left\lfloor \frac{q}{\sigma} \right\rfloor L + \mathrm{mod}\left(q, \sigma\right) \right] \Delta, \quad q \in \left[0, \sigma^2 - 1\right] \tag{3}$$

where Δ denotes the time delay for a symbol, i.e. $1/f_{clock}$. The number of delay steps is σ^2 in total. '$\lfloor\cdot\rfloor$' is the floor integer operation and 'mod' is the remainder operation. Suppl. Fig. 20 illustrates a convolution example with 3×3 kernel on a 10×10 image. As the convolutional kernel moves on the image, four steps of convolution are shown in Suppl. Fig. 20a. Inside a row, small delays should be used to synchronize the neighboring pixels. Since the convolutional kernel crosses three rows, long delays should be used to shift the pixels of the next two rows to the same temporal position of the first pixel. The structure to implement such optical delays is shown in Suppl. Fig. 20b. Unit delay shift the neighboring pixels and the long delay shifts the pixels across rows. In the example of 10×10 image, assuming that the clock frequency is 20 GHz, the required delays for different time dimensions are 0 ps, 50 ps, 100 ps, 0.5 ns, 0.55 ns, 0.6 ns, 1 ns, 1.05 ns, 1.1 ns, respectively. Since the delay lines in the PTFP chip is cascaded, the total length of the optical delay lines is determined by the maximal delay. The required length of optical delay lines is at meter-long level. Given that current technologies of low-loss silicon nitride waveguide support the insertion loss of 1.0 dB/m, long delay of optical signals is technically feasible. The precision of delay length is impactful to the accuracy of computing. Due to the fabrication error and the deviation of group

index during design, the row-level long ODLs may suffer from length imprecision. A typical solution is introducing tuning components such as microheaters [6, 7] to perform calibration for minor errors. Large errors should be optimized by changing designs and iterative trials. Advanced modulators and photodetectors with a large bandwidth allow us to operate the PTFP in higher clock rate: thus, a delay unit is shorter. The on-chip delay line is correspondingly shortened.

Besides the enhancement of functionality, larger integration scale also promises better energy efficiency of photonic processing. The analog photonic processing requires digital/analog interconversions and electrical/photonic interconversions. Compared with digital processors, the usage of DACs, ADCs, lasers, modulators, and photodiodes will consume extra power. As the number of integrated computing unit (MRR in this work) increases, the energy overhead by the interconversions will be diluted. Specifically, the FoM of DACs is formulated as $2^{bit}*fs/P$. A 28nm-CMOS process fabricated DAC has the specifications of 177 mW, 10 GS/s, and 14 bits [8]. Then, a DAC working at 20 GS/s and 8 bits is evaluated to consume the power of $177/2^{(14-8)}\times 2=5.53$ mW. For ADCs, several high-speed ADCs [9-11] are referenced, including a 5 GS/s ADC consuming 29 mW, a 10 GS/s ADC consuming 50.8 mW, and a 32 GS/s ADC consuming 199 mW. The power consumption grows approximately linearly with SNDR drops from 48.5 dB to around 43 dB. It infers that a 20 GS/s ADC may consume the power of ~120 mW at 7-bit effective resolution. This estimation is also consistent with the observation in a review of ADCs [12]. Then, the A/D interconversion of a symbol introduces (120+5.53)/20=6.28 pJ overhead. For lasers, the wall-plug efficiency is set to 5%. The laser power to support a C_{out}=32, D_{kernel}=9 convolutional kernel is evaluated as 7.96 W in total. We note that the SNR of photodetection is not influenced by the number of input channels with fixed input optical power. Ref. [4] presents the details. For modulators and PDs, the power budget of a symbol is evaluated at around 317 fJ [13]. Assume a [3×3, 32, 32] kernel is implemented on-chip (technically feasible), the energy overhead for a single operation can be calculated as

$$E=\left(\frac{(120+5.53)\ [\text{mW}]}{20\ \text{Gbaud}\times 32\times 9}+\frac{7960\ [\text{mW}]}{20\ \text{Gbaud}\times 32\times 32\times 9}+\frac{0.317\ [\text{pJ}]}{32*9}\right)/2=0.0329\ \text{pJ}. \tag{4}$$

where the first term is from the A/D interconversion; the second term is from the lasers; the third term is from the E/O interconversions.

Moreover, the PTFP follows the same rule as other photonic processors do. The flexibility of reconfiguration is lower than digital electronics and each round of reconfiguration consumes extra

tuning energy [14]. The way of reaching high efficiency is increasing the integration scale and enlarging the data batch size so that a large amount of computation is conducted within a round of reconfiguration.

Supplementary Note 7 - Throughput and compute density of the PTFP chip

Throughput of the chip is defined as the number of operations per second. The PTFP chip conducts multiply and accumulation (MAC) operations, corresponding to 2 operations (a multiplication and an addition) per clock cycle per unit. The throughput of the chip is therefore calculated by 12×20×2=480 GOP/s. Compute density describes the compute throughput in a unit area. Suppl. Fig. 21a and 21b provides the details of area measurement. There must be a separation between MRRs or the thermal crosstalk will be significant. In the PTFP chip, the separation is 0.12 mm, so the footprint of an MRR is 0.12×0.12=0.0144 mm^2. The on-chip delay line was originally designed as 100 ps, which serves for 10 Gbaud clock rate. The 100-ps ODL takes 0.65×0.495=0.3218 mm^2. The demonstrated PTFP chip comprises 12 MRRs and two 100-ps ODLs. The computing density of the photonic core is 480/(0.3218×2+0.0144×12)= 587.9 GOP/s/mm^2.

Given that the area efficiency can be significantly improved with more MRRs, in this part we envision the area efficiency with large-scale integration. To realize a two-dimensional convolutional kernel, row-level long ODLs should be integrated on-chip. Based on the silicon nitride platform, we design a long ODL covering 64-pixel delaying. The bending radius is set to 100 microns [15]. The layout is shown in Suppl. Fig. 21c. It occupies an area of 2.5*2.66=6.65 mm^2. Based on the area occupation values and the operation clock frequency (20 Gbaud), Suppl. Table 5 shows several examples of area occupation of ODLs and the computing density of the chip. The width (L) of the input image is set to 64. So, the area occupied by the Si_3N_4 ODLs is 15.2 mm^2, 33.0 mm^2, and 53.4 mm^2 for 3×3, 5×5, and 7×7 kernels, respectively. The number of input channels or wavelengths is set to 8, 16, and 32. The number of output channels is set to 32, 11, and 6 for 3×3, 5×5, and 7×7 kernels, respectively. It is seen that more MRRs integrated on the chip will dilute the area occupation of ODLs. Among all examples listed in Suppl. Table 5, the largest chip footprint is (53.4+64×7×7×6×0.0144)=324.3 mm^2, compatible with current photonic integration technologies.

Supplementary References

1. F. Horst, W. M. Green, S. Assefa, S. M. Shank, Y. A. Vlasov, and B. J. Offrein, Cascaded Mach-Zehnder wavelength filters in silicon photonics for low loss and flat pass-band WDM (de-) multiplexing. Optics Express **21**, 11652-11658 (2013).
2. X. Jiang, H. Wu, and D. Dai. Low-loss and low-crosstalk multimode waveguide bend on silicon. Optics Express **26**, 17680-17689 (2018).
3. A. N. Tait, Silicon photonic neural networks, Ph.D dissertation at Princeton university, 2018.
4. S. Xu, J. Wang, and W. Zou, Optical convolutional neural network with WDM-based optical patching and microring weighting banks, IEEE Photonics Technology Letters **33**, 89-92 (2021).
5. S. Xu, J. Wang, and W. Zou, Optical patching scheme for optical convolutional neural networks based on wavelength-division multiplexing and optical delay lines, Optics Letters 45, 3689-3692 (2020).
6. X. Wang, L. Zhou, R. Li, J. Xie, L. Lu, K. Wu, and J. Chen, Continuously tunable ultra-thin silicon waveguide optical delay line, Optica **4**, 507-515 (2017).
7. X. Ji, X. Yao, Y. Gan, A. Mohanty, M. A. Tadayon, C. P. Hendon, and M. Lipson, On-chip tunable photonic delay line, APL Photonics **4**, 090803 (2019).
8. C. Demirkiran, F. Eris, G. Wang, J. Elmhurst, N. Moore, N. C. Harris, A. Basumallik, V. J. Reddi, A. Joshi, D. Bunandar, An electro-photonic system for accelerating deep neural networks, preprint at arXiv: arXiv:2109.01126v1 (2021).
9. M. Guo, J. Mao, S. Sin, H. Wei, and R. Martins, A 5 GS/s 29 mW interleaved SAR ADC with 48.5 dB SNDR using digital-mixing background timing-skew calibration for direct sampling applications, IEEE Access **8**, 138954 (2020).
10. M. Zhang, Y. Zhu, C. Chan, and R. Martins, An 8-Bit 10-GS/s 16× interpolation-based time-domain ADC with <1.5-ps uncalibrated quantization steps, IEEE Journal of Solid-State Circuits **55**, 3225-3235 (2020).
11. L. Kull, D. Luu, C. Menolfi, T. Morf, P. Francese, M. Braendli, M. Kossel, A. Cevrero, I. Ozkaya, and T. Toifl, A 10-bit 20-40 GS/s ADC with 37 dB SNDR at 40 GHz input using first order sampling bandwidth calibration, IEEE Symposium on VLSI Circuits (2018).
12. B. Murmann, The race for the extra decibel: a brief review of current ADC trajectory, IEEE Solid-State Circuit Magazine **7**, 58-66 (2015).

13. C. Sun, M. Wade, Y. Lee, J. Orcutt, L. Alloatti, M. Georgas, A. Waterman, J. Shainline, R. Avizienis, S. Lin, B. Moss, R. Kumar, F. Pavanello, A. Atabaki, H. Cook, A. J. Ou, J. Leu, Y. hsin Chen, K. Asanovi´c, R. J. Ram, M. Popovic, and V. Stojanovi´c, Single-chip microprocessor that communicates directly using light, Nature **528**, 534-538 (2015).
14. M. A. Al-Qadasi, L. Chrostowski, B. J. Shastri, and S. Shekhar, Scaling up silicon photonic-based accelerators: Challenges and opportunities, APL Photonics **7**, 020902 (2022).
15. X. Ji, F. A. S. Barbosa, S. P. Roberts, A. Dutt, J. Cardenas, Y. Okawachi, A. Bryant, A. L. Gaeta, and M. Lipson, Ultra-low-loss on-chip resonators with sub-milliwatt parametric oscillation threshold, Optica **4**, 619-624 (2017).
16. N. P. Jouppi, C. Young, N. Patil, D. Patterson, G. Agrawal, R. Bajwa, S. Bates, S. Bhatia, N. Boden, A. Borchers, R. Boyle, P. Cantin, C. Chao, C. Clark, J. Coriell, M. Daley, M. Dau, J. Dean, B. Gelb, T. V. Ghaemmaghami, R. Gottipati, W. Gulland, R. Hagmann, C. R. Ho, D. Hogberg, J. Hu, R. Hundt, D. Hurt, J. Ibarz, A. Jaffey, A. Jaworski, A. Kaplan, H. Khaitan, D. Killebrew, A. Koch, N. Kumar, S. Lacy, J. Laudon, J. Law, D. Le, C. Leary, Z. Liu, K. Lucke, A. Lundin, G. MacKean, A. Maggiore, M. Mahony, K. Miller, R. Nagarajan, R. Narayanaswami, R. Ni, K. Nix, T. Norrie, M. Omernick, N. Penukonda, A. Phelps, J. Ross, M. Ross, A. Salek, E. Samadiani, C. Severn, G. Sizikov, M. Snelham, J. Souter, D. Steinberg, A. Swing, M. Tan, G. Thorson, B. Tian, H. Toma, E. Tuttle, V. Vasudevan, R. Walter, W. Wang, E. Wilcox, and D. H. Yoon, In-datacenter performance analysis of a tensor processing unit, Proceedings of the 44th annual international symposium on computer architecture, 1-12 (2017).

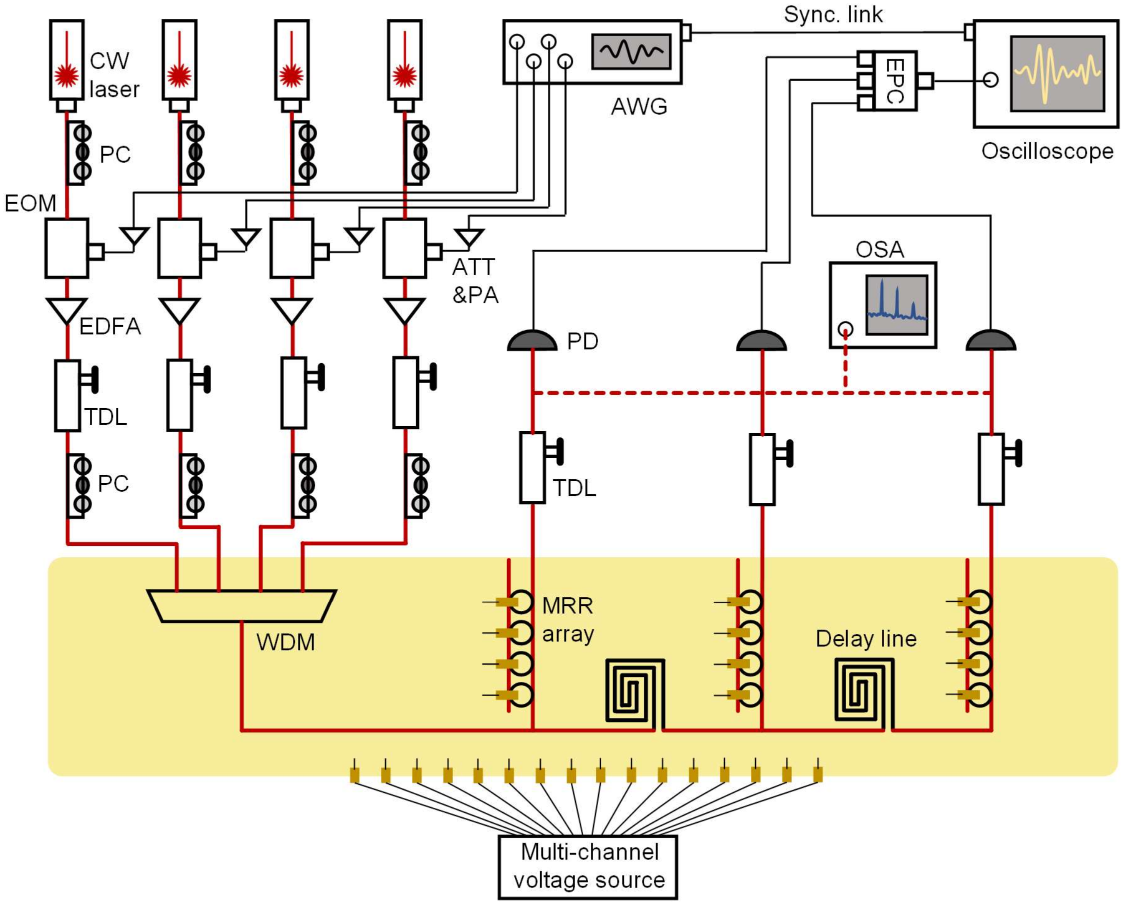

Suppl. Fig. 1 Experimental setup of photonic tensor flow processing. PC, polarization controller; EOM, electrooptic modulator; EDFA, erbium-doped fiber amplifier; TDL, tunable delay line; ATT, attenuator; PA, power amplifier; AWG, arbitrary waveform generator; WDM, wavelength division multiplexer; OSA, optical spectrum analyzer; EPC, electrical power combiner.

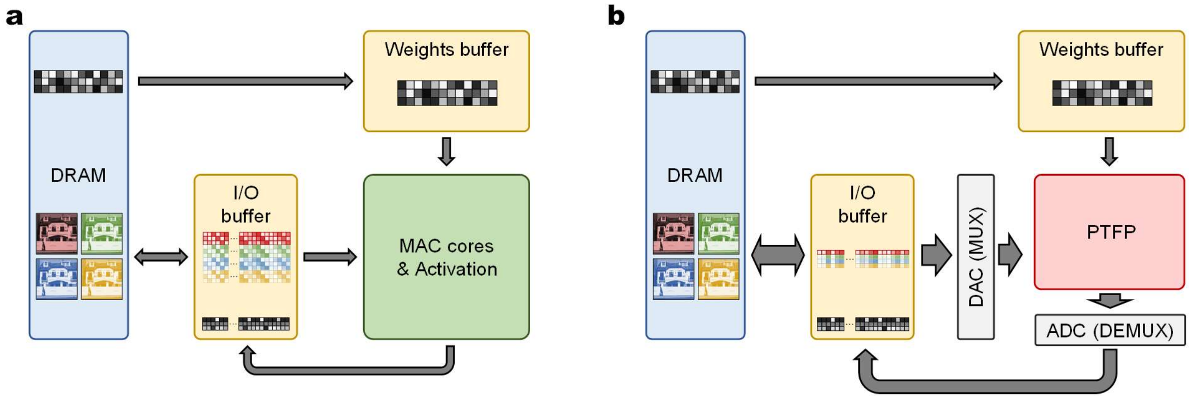

Suppl. Fig. 2 Circuitry of conventional digital GeMM and the PTFP. a, Brief circuitry of Google's tensor processing unit (TPU), extracted from ref. [16]. To deal with GeMM, the input data should be firstly duplicated in the DRAM or the I/O buffer (typically SRAM). Then the buffered data enters the MAC cores for matrix computing. Weights are loaded to the MAC cores via a weights buffer. **b,** The envisioned circuitry of the PTFP. The input data is moved to the I/O buffer and then converted to analog signals through the DAC. Since data duplication is not required, the throughput of data movement is only limited by the memory interface width. The DACs work in a time multiplexing, converting parallel low-speed electronic signals to serial high-speed optical signals. At the output of the photonic chip, ADCs work in a time demultiplexing manner, converting serial high-speed outputs to parallel low-speed digital data for storage.

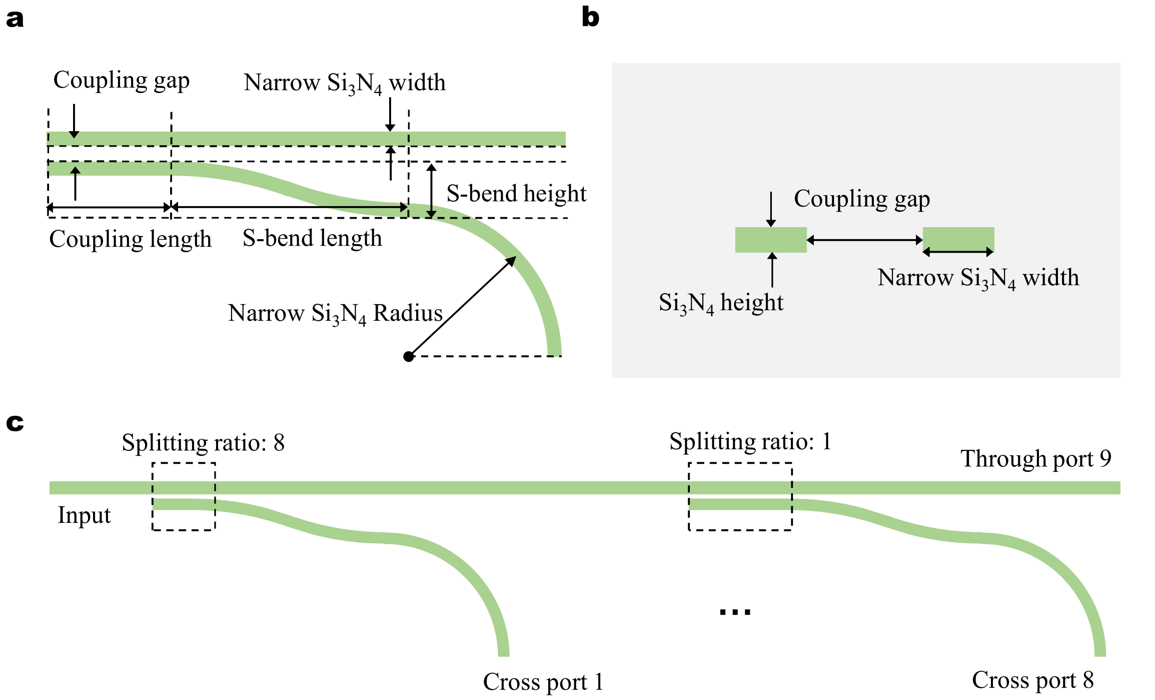

Suppl. Fig. 3 Si_3N_4 directional coupler. a, The schematic of directional coupler and **b**, the cross-section of the two separating waveguides. **c**, The cascaded DCA with different splitting ratio, ranging from 8:1 to 1:1. Cross ports are labeled from 1 to 8, and the through port is the nineth port.

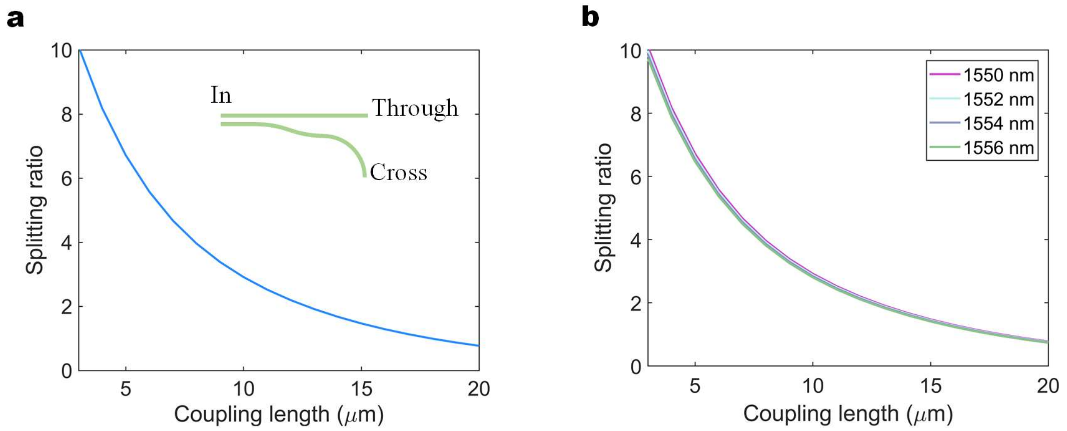

Suppl. Fig. 4 Simulation results of Si_3N_4 directional coupler. a, The splitting ratio between the through port and the cross port at 1550 nm, and **b**, wavelength dependence of the splitting ratio.

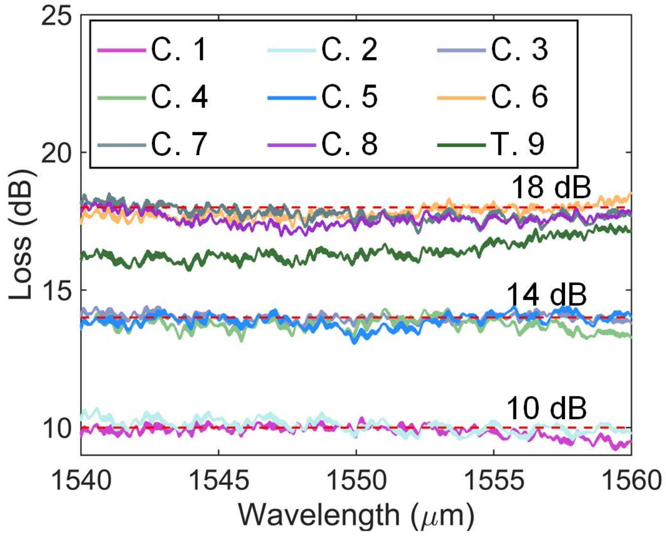

Suppl. Fig. 5 Measured insertion loss of cross port over 1540~1560 nm. C.1-C.8 represent the insertion loss of cross port 1-8, respectively. T. 9 represents the insertion loss of the through port. It is shown that there is a large unevenness between these output ports which are designed to be uniform splitting. The device should be optimized in future works.

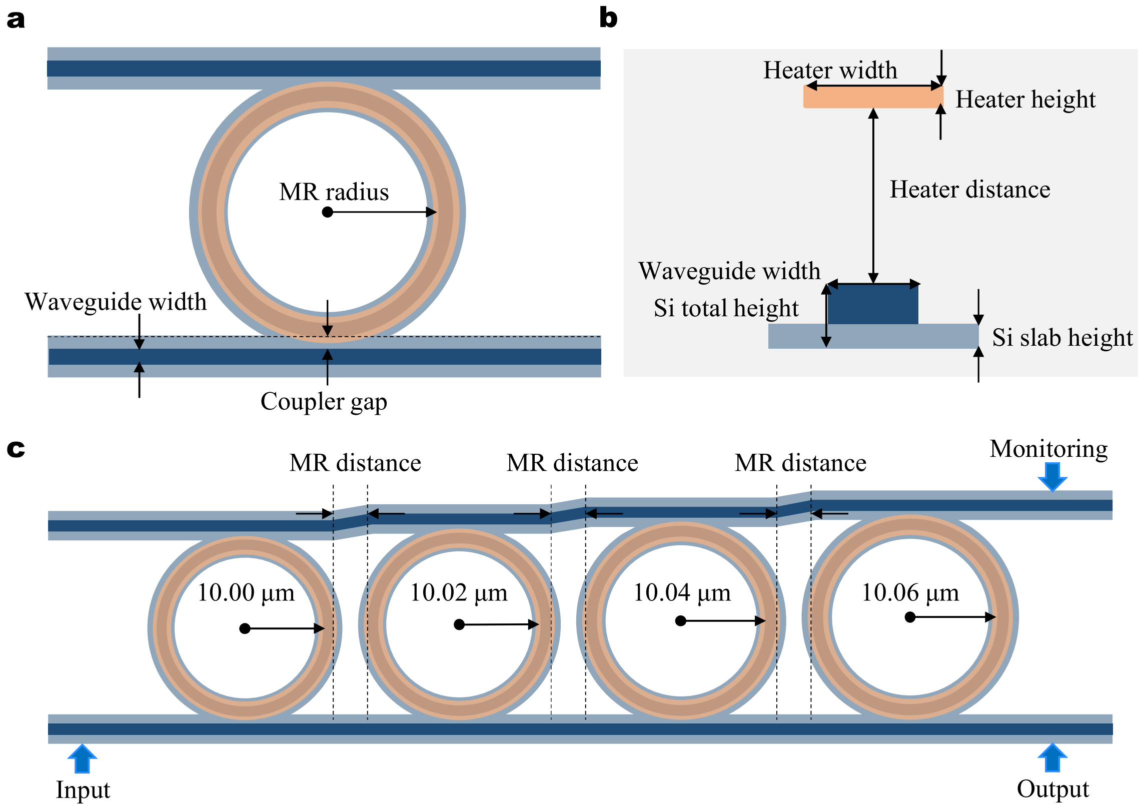

Suppl. Fig. 6 Tunable Silicon MRR weight bank. a, The schematic of the Si micro-ring and **b**, the cross-section of the Si waveguide and micro heater. **c**, Detailed design schematic of the micro-ring weight bank

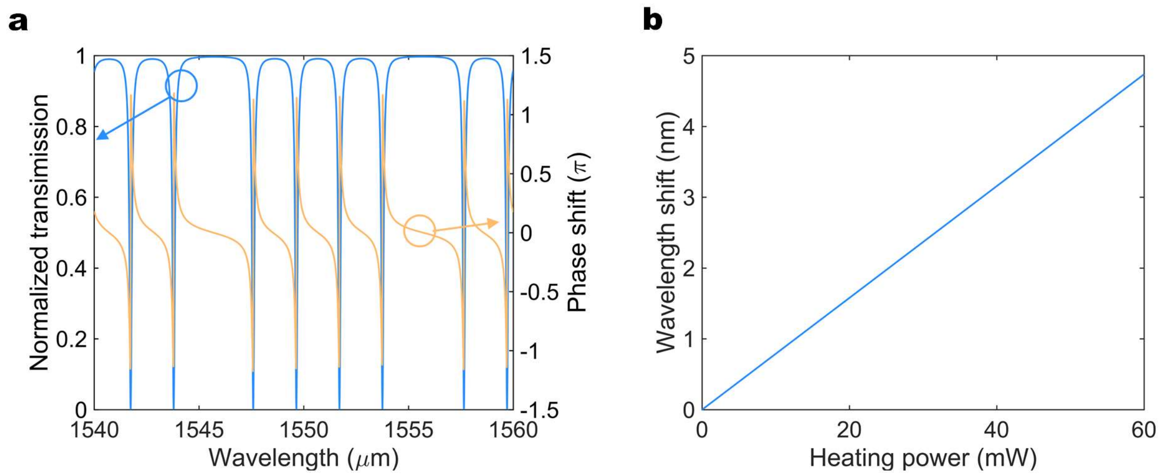

Suppl. Fig. 7 Simulation results of the MRR weighting bank. a, Calculated transmission and phase responses of the tunable Si micro-ring weight bank. **b**, Simulated wavelength shift as a function of the heating power with the tuning efficiency of 78.9 pm/mW.

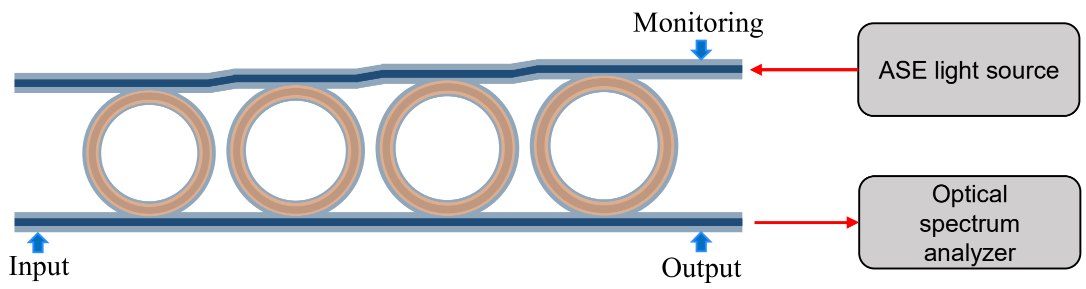

Suppl. Fig. 8 Measurement setup of the MRR weighting bank. ASE light source provides a wideband optical spectrum and the optical spectrum analyzer inspects the transmission of the fabricated MRRs. With different tuning voltages, different transmission curves are obtained and shown in Fig. 2f. Since the monitoring port is coupled to the output port via MRR resonating, the transmission curves between the input and output port should be complementary to the measured curves.

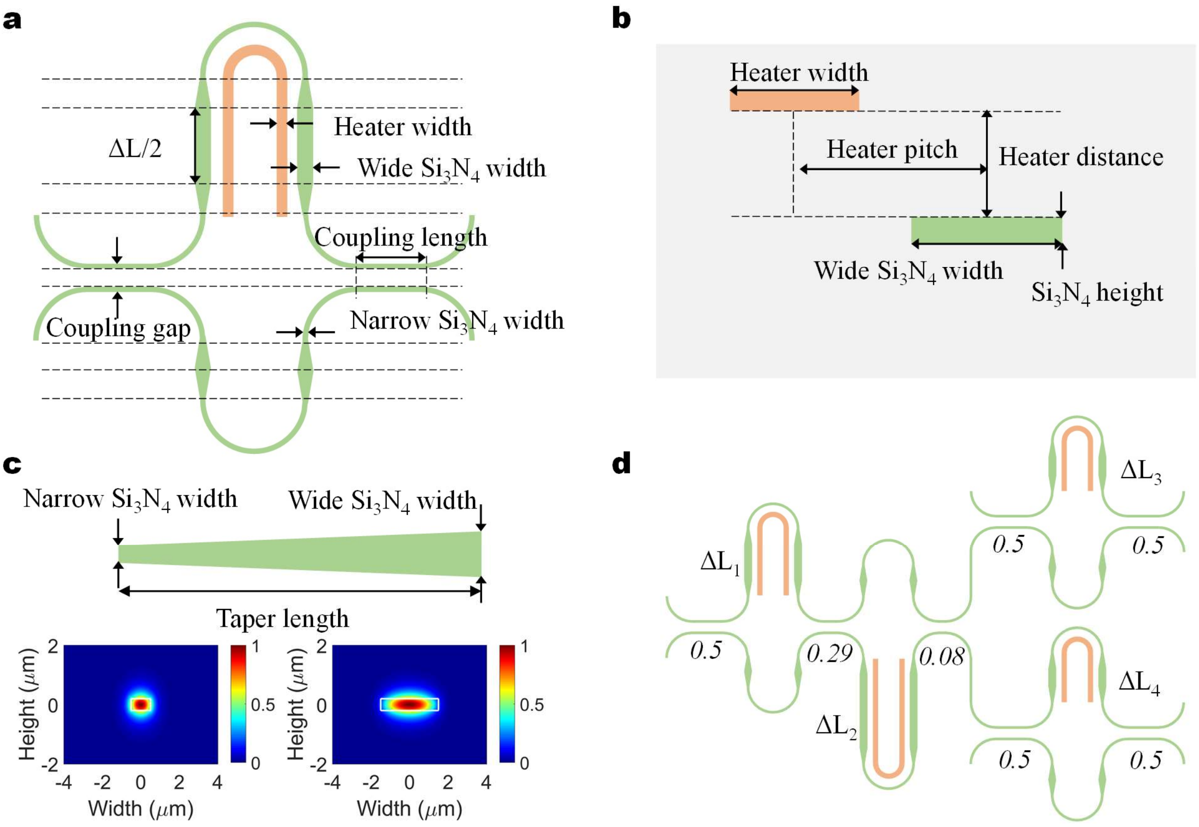

Suppl. Fig. 9 Tunable Cascaded Mach-Zehnder WDM. a, The schematic of the SiN phase shifter and **b,** the cross-section of the SiN waveguide and micro heater. **c,** The waveguide taper between the wide waveguide and the narrow waveguide. The simulated transverse electric (TE) field profile of fundamental mode in the narrow waveguide (left) and in the wide waveguide (right). **d,** Detailed design schematic of the four channel WDM, 0.5, 0.29, and 0.08 are the power cross-coupling coefficients of the 2×2 directional couplers, and ΔL_i denotes the length of the wide waveguide of the i-th MZI.

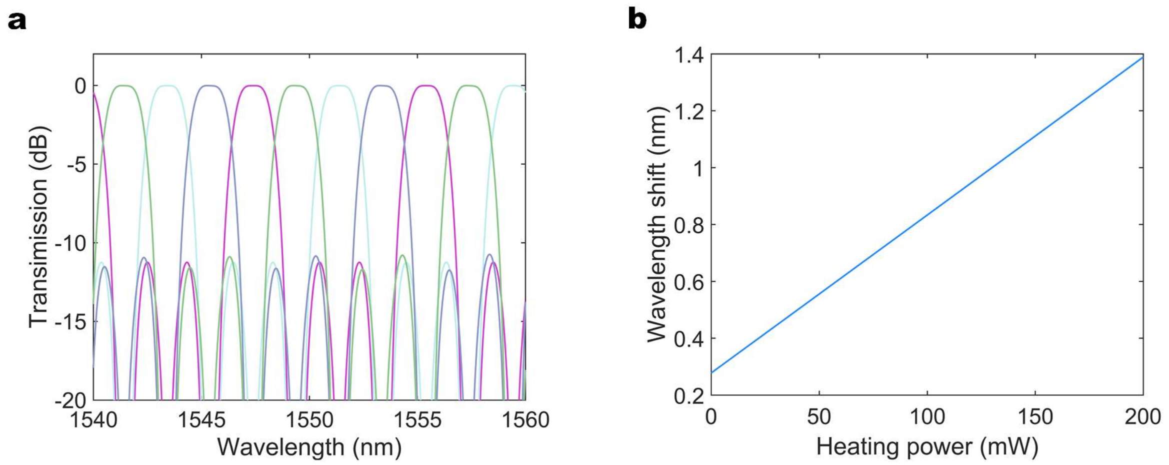

Suppl. Fig. 10 Simulation results of the WDM. a, Calculated transmission and phase responses of the 4-channel WDM. **b**, Simulated wavelength shift as a function of the heating power, while the tuning efficiency is 5.6 pm/mW.

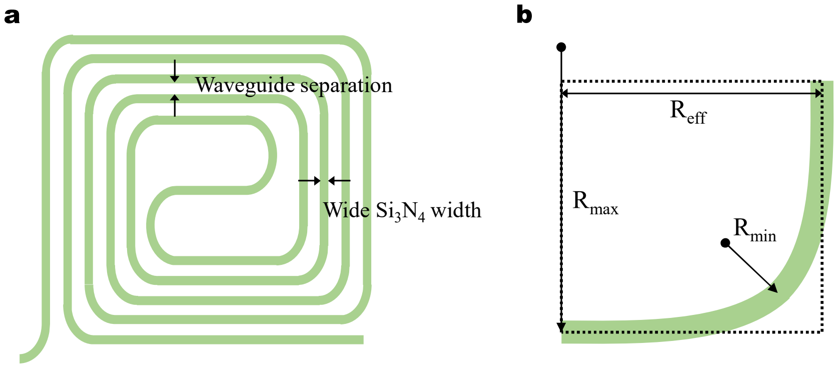

Suppl. Fig. 11 Schematic of Si_3N_4 delay line. a, The schematic of the Si3N4 delay line and **b**, the 90º Euler bend.

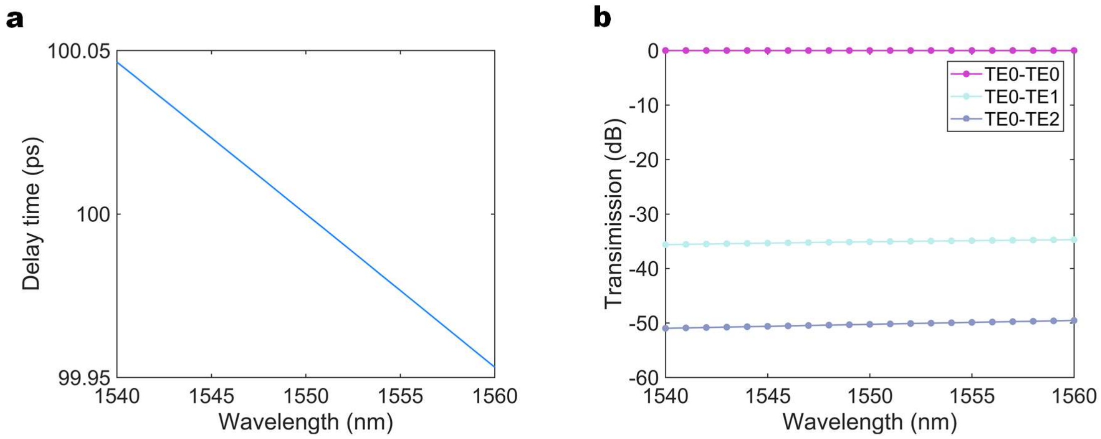

Suppl. Fig. 12 Simulation results of the delay line. **a**, Wavelength dependence of the group delay. **b**, Calculated transmission from the fundamental TE mode launched at the input to the *i*-th TE mode in the output.

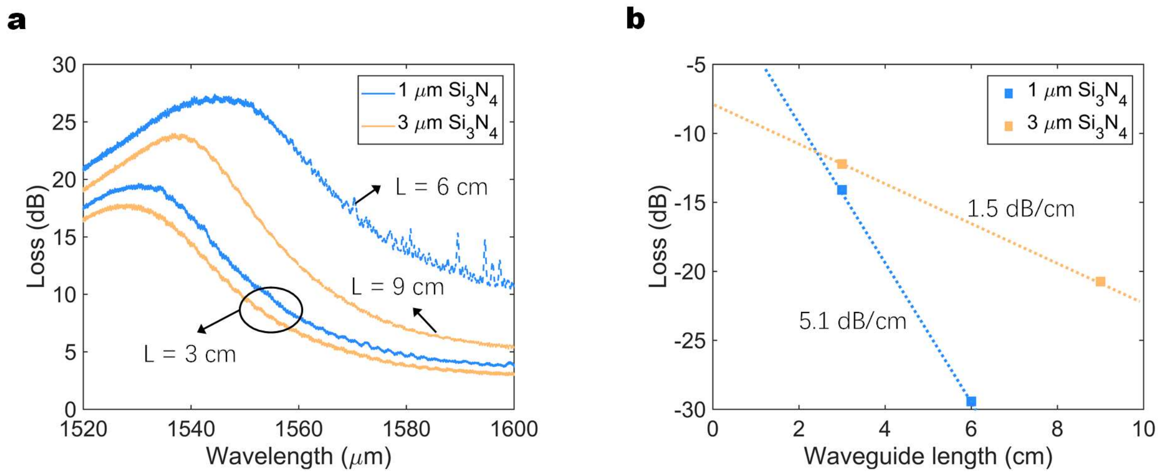

Suppl. Fig. 13 Measured results of the delay lines of different widths. a, Measured transmission spectra of the Si3N4 delay line with different widths. **b**, Measured loss of the Si_3N_4 delay lines with different width at 1550 nm. The loss of the 1-μm Si_3N_4 waveguide is 5.1 dB/cm, while that of the 3-μm Si_3N_4 waveguide is around 1.5 dB/cm.

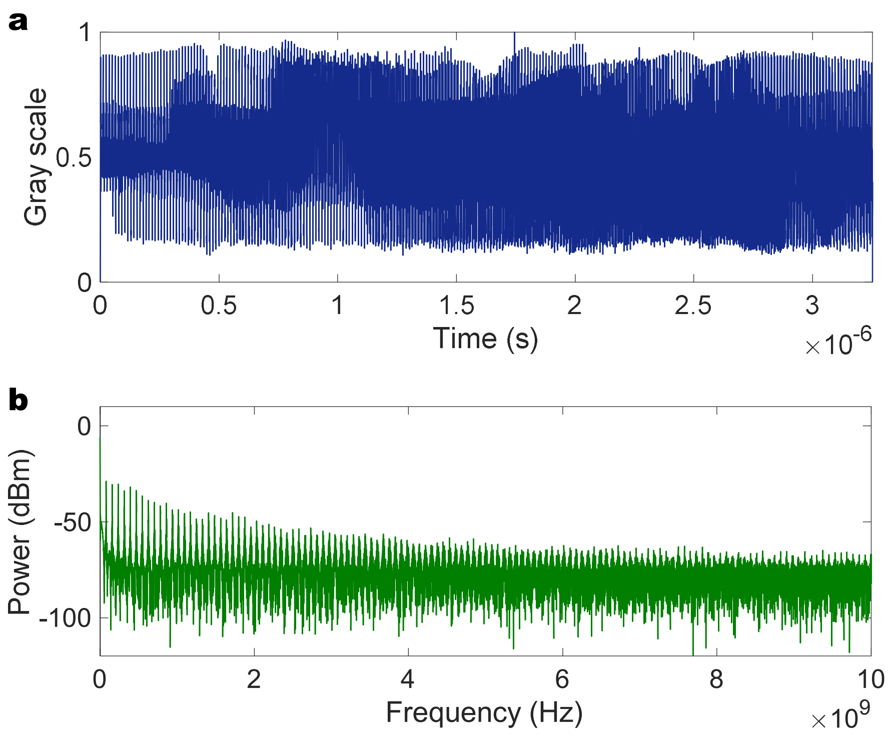

Suppl. Fig. 14 Temporal waveform (a) and the frequency spectrum (b) of the vectorized image. The original image is the gray-scaled 'Lena'. Image is reshaped to a row vector to show the temporal waveform. The average gray-scale value is around 0.5, indicating that power at DC component is large. For other natural images, the waveform and spectrum may change but the distribution is similar: large low-frequency components and small high-frequency components.

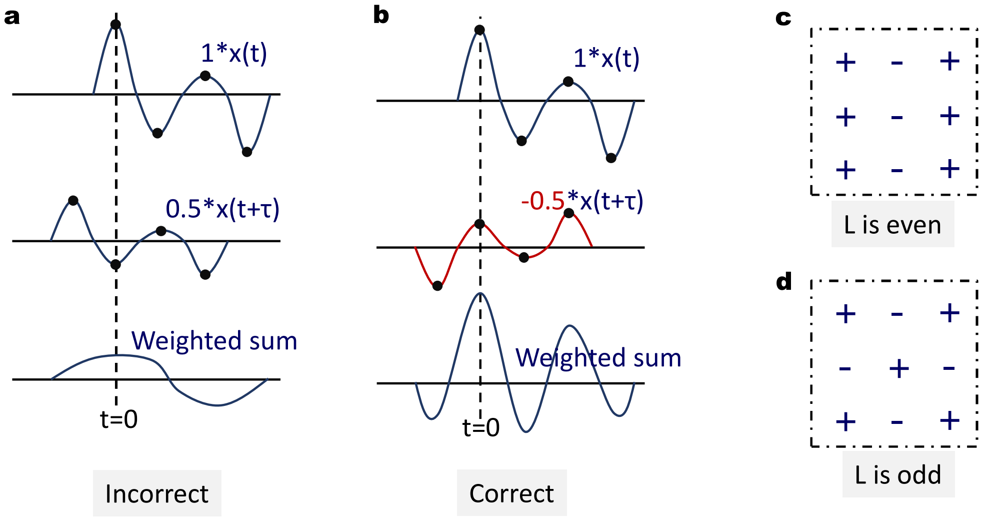

Suppl. Fig. 15 Encoding method of convolutional kernels. Suppose a kernel [1, 0.5] should be implemented. **a**, The kernel is not encoded so that the implemented kernel is actually [1, -0.5]. **b**, The kernel is firstly encoded to [1, -0.5] so that the implemented kernel is actually [1, 0.5]. Encoding mask for 3×3 kernels when the length of image row is even (**c**) and odd (**d**).

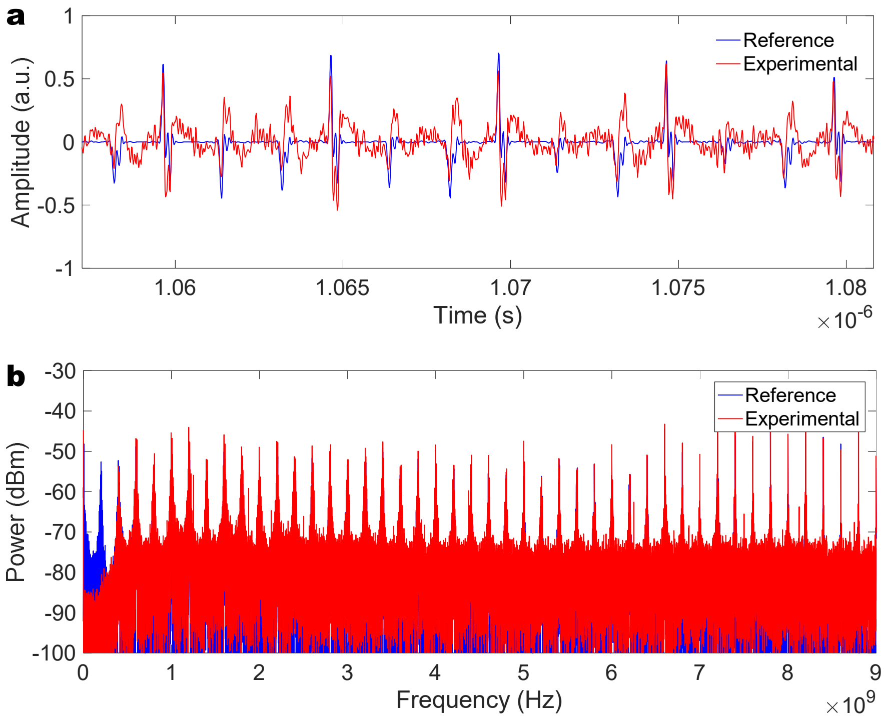

Suppl. Fig. 16 Temporal waveform (a) and frequency spectrum (b) of the convolved image. The applied kernel is [-1, 0, 1]. Reference is the ideal result of the convolution. Due to the lack of low-frequency part, the temporal waveform is distorted.

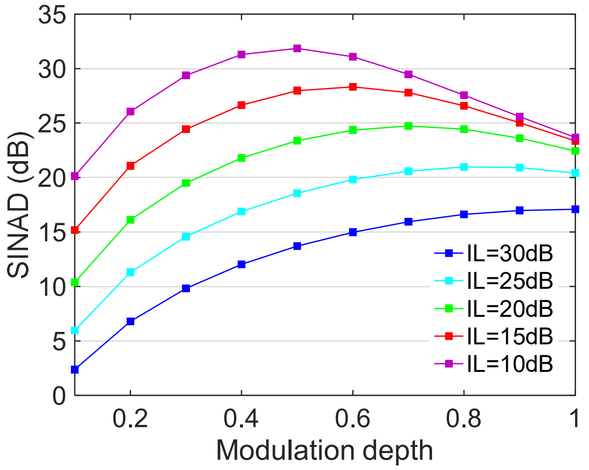

Suppl. Fig. 17 Signal quality (SINAD) with different modulation depth and insertion loss. The result is simulated with identical conditions in the experiment by assuming different insertion losses. 'IL=30 dB' represents the performance of the current photonic chip. The output signal quality can be refined by both higher modulation depth and better loss performance. In the cases that IL is low, too large modulation depth leads to a signal quality degradation because of the nonlinearity of electro-optic modulation.

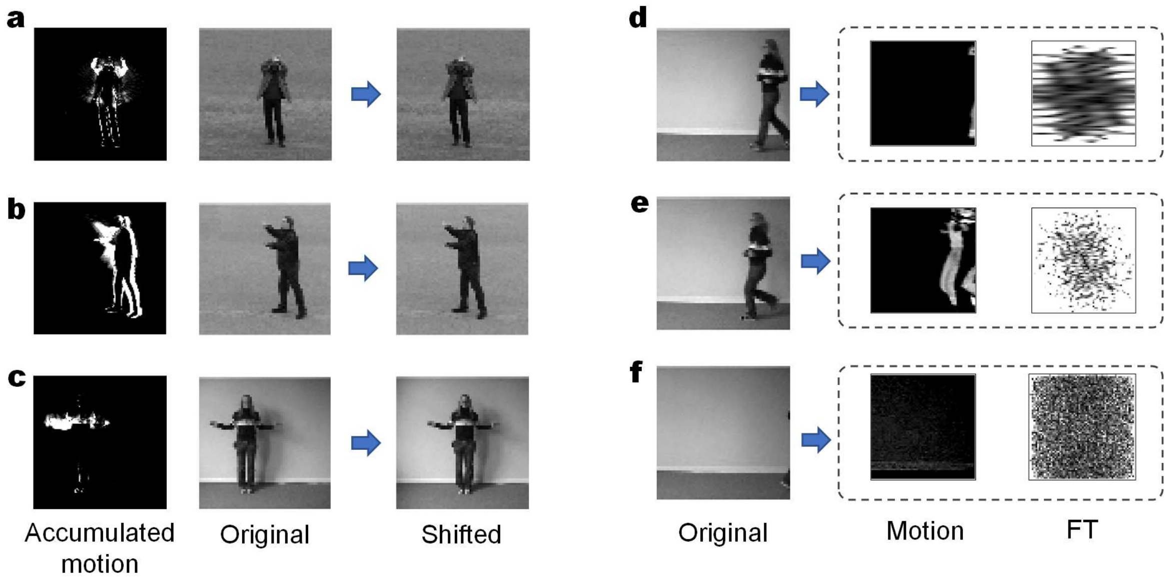

Suppl. Fig. 18 Preprocessing method for the KTH dataset. a-c, Methods to process static actions. Accumulated motion is obtained by accumulating the pixel variation during the video. The video is shifted to assure that the subject locates at the image center. **d-f**, Methods to process moving actions. **d**, If the subject does not enter the central part of the image, the motion detection turns out a result below the threshold. **e**, The subject enters the central part of the image, and motion detection turns out a result of valid motion. **f**, The moving or shaking of the camara itself will turn out a valid motion, because of the background. But the Fourier transform ('FT') of the motion is white-noise-alike.

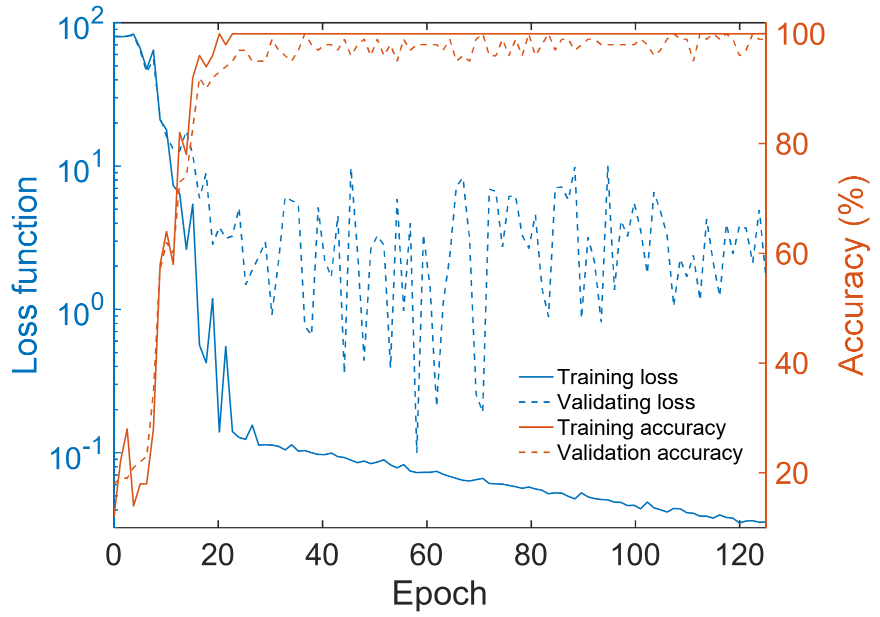

Suppl. Fig. 19 Loss functions and accuracies during training. Training loss is calculated by the average loss in the training batch (batch size = 50, randomly selected from the trainset). Validation loss is calculated from the validation batch (batch size = 100, randomly selected from the testset). The training loss decreases consistently and reaches a very small value. It reflects that the convolutional neural network can fit the trainset very well. The validation loss shows that the generalizability of the CNN is limited. It may be limited by the network model itself or the inappropriate preparation of datasets. From the validation accuracy curve, the training performance (~99%) is acceptable.

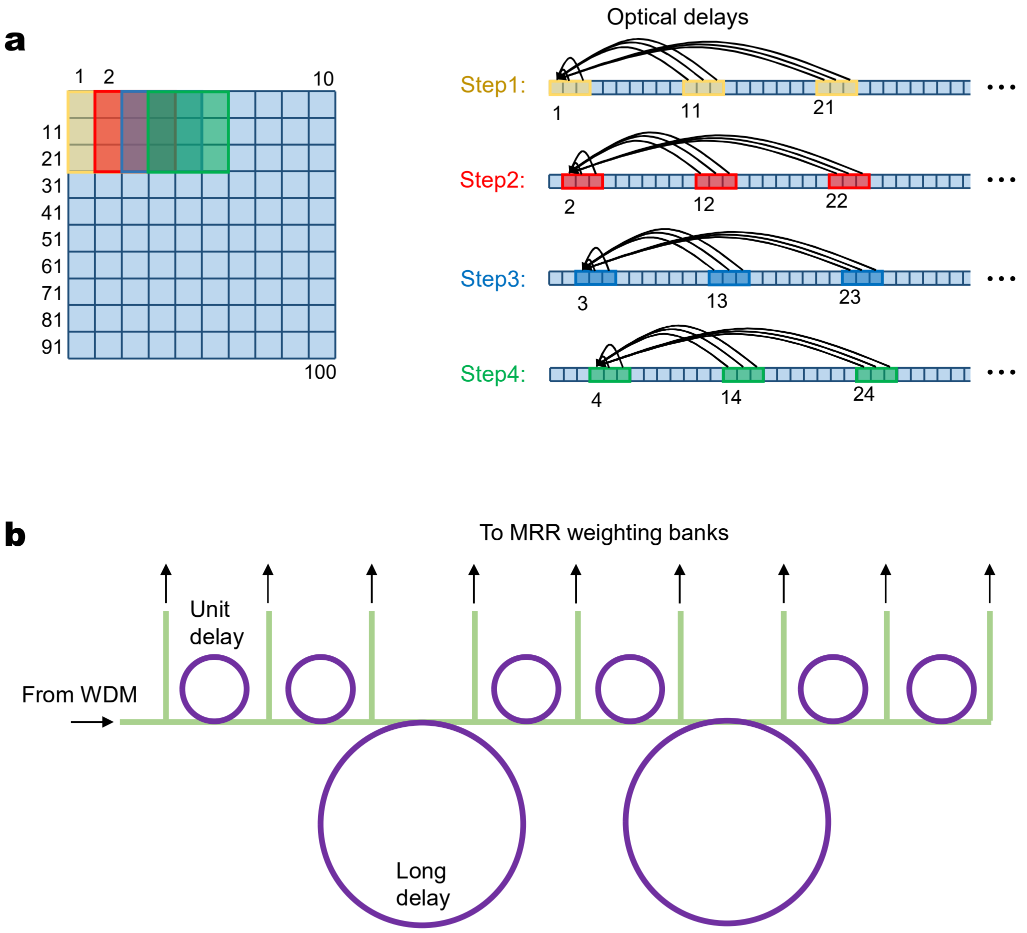

Suppl. Fig. 20 The method of implementing 3×3 kernel with optical delays. The image is converted to a vector row by row. **a**, Four steps of convolution are given. The optical delay structure should align the pixels covered by the kernel to the same time step. Therefore, the required delay amounts for different pixels are not uniform. **b**, The optical delay structure. Unit delay accomplishes the alignment for adjacent pixels and long delay is for adjacent rows.

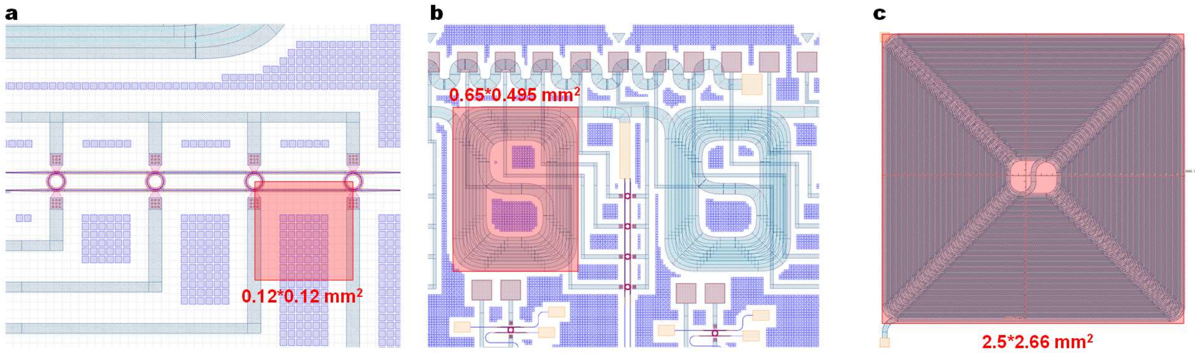

Suppl. Fig. 21 Layout of the MRRs (a) and the optical delay line (b) fabricated on-chip. The footprint of MRRs is evaluated as the separation of MRRs. (c) The layout of a long ODL covering 64-pixel time delay. It is designed with silicon nitride platform and 100-micron bending radius.

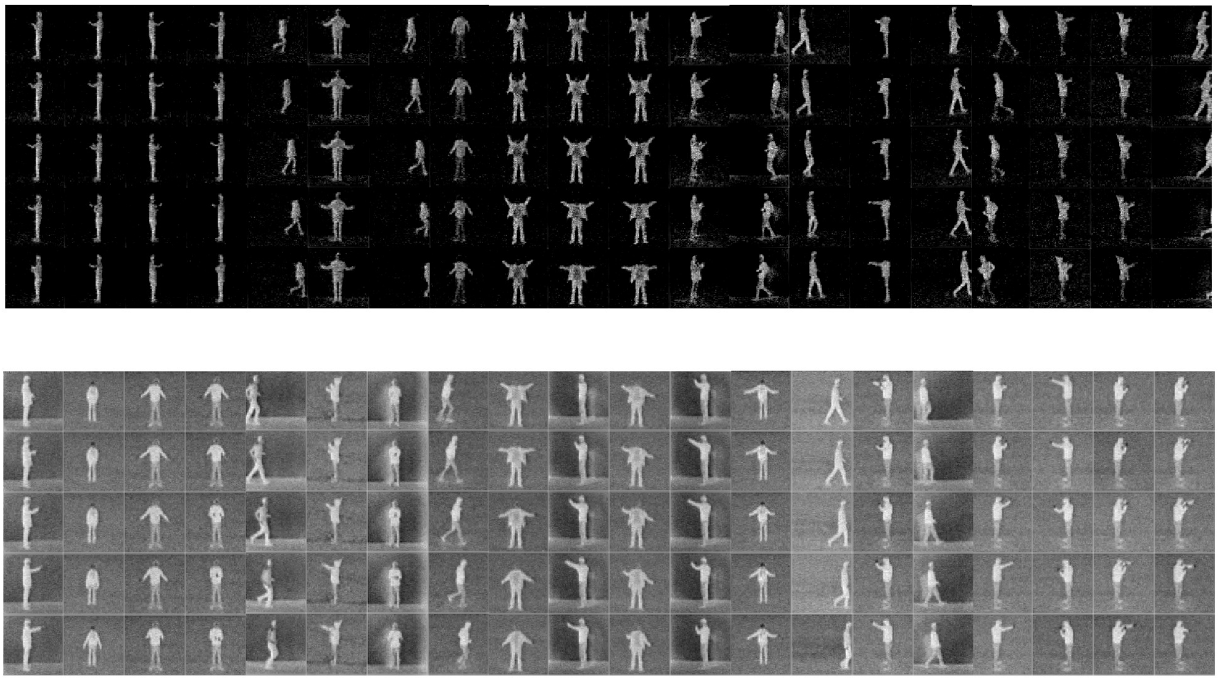

Suppl. Fig. 22 Convolved results of the first convolutional layer (conv. 1). Example results of two output channels (Ch.1, and Ch.2) are shown. The amplitudes of Ch.3 and Ch.4 are small, thus being excluded from the figure.

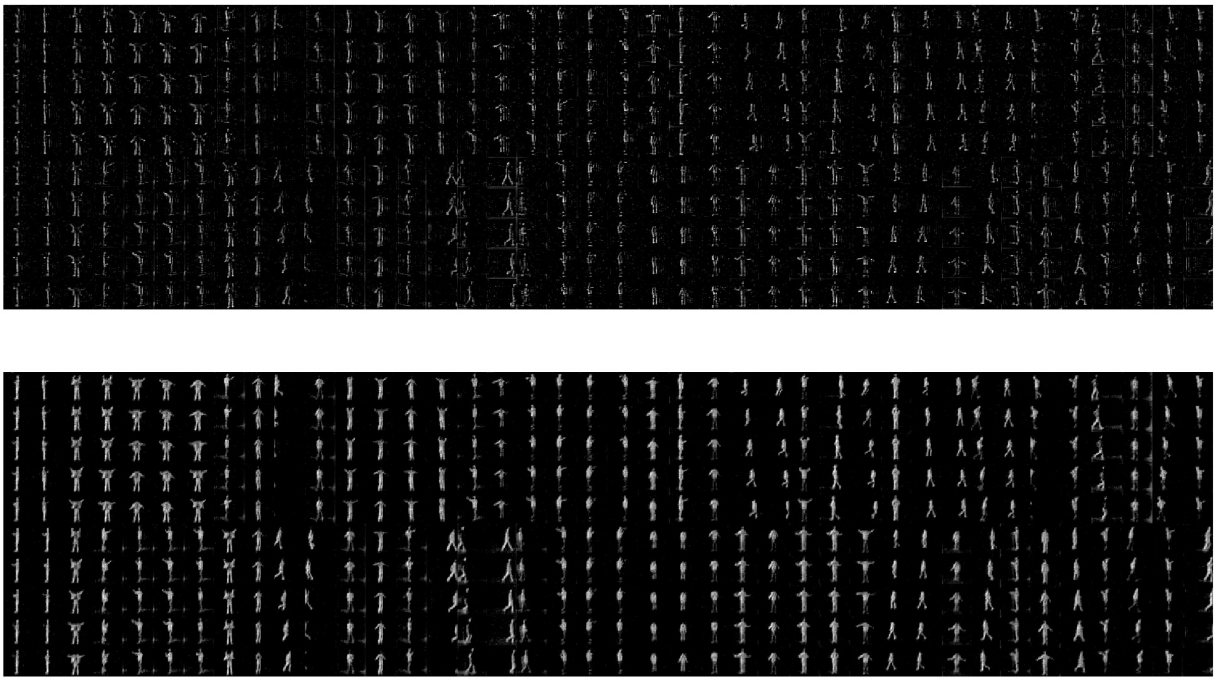

Suppl. Fig. 23 Convolved results of the second convolutional layer (conv.2). Example results of output Ch. 4 and output Ch. 8 are shown. Other channels also agree well with the reference results.

Suppl. Table 1 Geometrical parameters (in μm) for the Si_3N_4 directional couplers. The S-bend height is 4 mm in fabrication.

Coupling gap	0.45
S-bend length	3
S-bend height	30
Narrow Si_3N_4 Radius	60
Narrow Si_3N_4 width	1
Narrow Si_3N_4 width	0.4

Suppl. Table 2. The coupling length (in µm) for the Si_3N_4 directional couplers of certainly splitting ratio between the through port and the cross port at 1550 nm.

Splitting ratio	1	2	3	4
Coupling length	17.90	12.68	9.80	7.93
Splitting ratio	5	6	7	8
Coupling length	6.62	5.62	4.80	4.15

Suppl. Table 3 Geometrical parameters (in µm) for the tunable Si micro-ring weight bank.

Si slab height	0.7	MR distance	100
Si total height	0.22	Heater width	2
Coupler gap	0.24	Heater height	0.12
Waveguide width	0.5	Heater distance	2000

Suppl. Table 4 Geometrical parameters (in µm) for the 4-channel MZI-type WDM.

Si_3N_4 height	0.40	Coupling gap	0.45
Narrow Si_3N_4 width	1.00	Taper length	50
Wide Si_3N_4 width	3.00	Narrow Si_3N_4 radius	60
Heater width	2.00	ΔL_1	301.90
Heater height	0.12	ΔL_2	603.80
Heater distance	1350	ΔL_3	150.95
Heater pitch	4.00	ΔL_4	151.74

Suppl. Table 5 The area of on-chip Si_3N_4 ODLs and overall computing density with different convolutional kernel sizes. ‘16λ’ means 16 wavelengths (input channels) are integrated on-chip. The number of MRRs is calculated by the product of input channel number, output channel number, and the kernel size.

Kernel size	Area of on-chip delay lines [mm^2]	On-chip computing density [TOPS/mm^2]		
		8λ	16λ	32λ
3×3	15.2	1.91	2.26	2.49
5×5	33.0	1.36	1.82	2.20
7×7	53.4	1.08	1.55	1.99
